# Supplementary material for: Deletion of wfs1 Impairs Oligodendrocyte Precursor Cells Dorsal Distribution and Myelination Through the wfs1-hmgcs1 Axis in Zebrafish
Source: Biology (Basel). 2026 Aug 16;15(16):1402. doi: 10.3390/biology15161402 (PMC13510151; doi:10.3390/biology15161402)
Supplement: Supplementary file 1 [file biology-15-01402-s001.zip › File S1-Transcriptome sequencing data and differentially expressed genes (DEGs) in wfs1-- zebrafish at 5 dpf/2.KEGG/wfs1KOvsWT/ALL/src/dre04145.html]

dre04145


- dre:564266

- Up regulated genes

564266(2.42311476837666)

- dre:100004793

- Up regulated genes

100004793(6.25595816430424)
- dre:100537272

- Up regulated genes

100537272(1.07395112076968)

- dre:57934

- Up regulated genes

57934\_1(9.91304974129429)

- Down regulated genes

57934(-1.25539807424297)

- dre:403014

- Up regulated genes

403014(5.17135330153499)

- dre:792699

- Down regulated genes

792699(-1.84389282152821)

- dre:403014

- Up regulated genes

403014(5.17135330153499)

- dre:792699

- Down regulated genes

792699(-1.84389282152821)

- dre:431777

- Up regulated genes

431777(1.35055689210441)

- dre:336681

- Down regulated genes

406811\_1(-1.09337424046752)

- dre:359840

- Up regulated genes

359840(3.81608997342262)

- Down regulated genes

359840\_1(-1.77685900658053)
- dre:359839

- Up regulated genes

359839(1.49784899389719)

- dre:359840

- Up regulated genes

359840(3.81608997342262)

- Down regulated genes

359840\_1(-1.77685900658053)
- dre:359839

- Up regulated genes

359839(1.49784899389719)

- dre:571584

- Down regulated genes

571584(-1.08054144451958)

- dre:403131

- Down regulated genes

403131(-4.42862381698358)

- dre:100007982

- Down regulated genes

100007982(-1.68529326567621)

- dre:100005358

- Down regulated genes

100005358(-3.40764150774661)

- dre:30762

- Up regulated genes

30762(1.51549567324805)
- dre:368878

- Up regulated genes

368878(2.50701188401484)
- dre:30763

- Up regulated genes

30763(5.58822372755117)
- dre:571282

- Up regulated genes

571282(1.90251155561911)

- dre:30762

- Up regulated genes

30762(1.51549567324805)
- dre:368878

- Up regulated genes

368878(2.50701188401484)
- dre:30763

- Up regulated genes

30763(5.58822372755117)
- dre:571282

- Up regulated genes

571282(1.90251155561911)

- dre:556826

- Up regulated genes

556826\_1(2.25777649220643)

- dre:556826

- Up regulated genes

556826\_1(2.25777649220643)

- dre:359840

- Up regulated genes

359840(3.81608997342262)

- Down regulated genes

359840\_1(-1.77685900658053)
- dre:359839

- Up regulated genes

359839(1.49784899389719)

Close
